# Nanorods on surface of GaN-based thin-film LEDs deposited by post-annealing after photo-assisted chemical etching

**DOI:** 10.1186/s11671-016-1817-7

**Published:** 2017-01-13

**Authors:** Lung-Chien Chen, Wun-Wei Lin, Te-Yu Liu

**Affiliations:** Department of Electro-optical Engineering, National Taipei University of Technology, 1, Sec.3, Chung-Hsiao E. Rd, Taipei, 106 Taiwan

**Keywords:** Photo-enhanced post-annealing, Laser lift-off, KOH, Nanorods

## Abstract

This study investigates the optoelectronic characteristics of gallium nitride (GaN)-based thin-film light-emitting diodes (TF-LEDs) that are formed by a two-step transfer process that involves wet etching and post-annealing. In the two-step transfer process, GaN LEDs were stripped from sapphire substrates by the laser lift-off (LLO) method using a KrF laser and then transferred onto ceramic substrates. Ga-K nanorods were formed on the surface of the GaN-based TF-LEDs following photo-assisted chemical etching and photo-enhanced post-annealing at 100 °C for 1 min. As a result, the light output power of GaN-based TF-LEDs with wet etching and post-annealing was over 72% more than that of LEDs that did not undergo these treatments.

## Background

Flip-chip LEDs have been extensively utilized for backlighting in LCD televisions because they effectively increase the intensity of back-light source and reduce the cost of fabrication from LCD [1–5]. In the past, their use has been limited by their high unit price. The aims of the experimental stage are high reliability and low equipment cost. However, with the evolution of metal electrode materials and constant improvement in relevant processes, flip-chip LED technology has matured into something quite different from traditional LED technology [6–10]. Recently, thin film LEDs have attracted considerable attention as components in light sources for portable electronic devices because they are flat.

Gallium nitride (GaN)-based LEDs are stripped from sapphire substrates using the laser lift-off (LLO) method because sapphire exhibits poor heat dissipation. The substrates are fabricated by utilizing wafer bonding (or electroplating) and LLO methods [11–15]. After sapphire substrate has been removed from the GaN epitaxial layer, the exposed GaN surface is roughened by chemical etching using dilute aqueous KOH to improve its light extraction efficiency [16–20]. In this study, after wet etching and post-annealing at 100 °C for 1 min, nanorods grew on the surface of n-GaN. The hexagonal pyramid structure differed from that generally obtained using the KOH wet etching method. Finally, the optoelectronic characteristics of the GaN-based thin-film light-emitting diodes (TF-LEDs) that were fabricated with various etching times were examined.

## Methods

The GaN-based LED epitaxial wafer consisted of a 3-μm-thick GaN:Si layer, seven pairs of undoped InGaN/GaN multiple quantum wells, and a 0.25-μm-thick layer of GaN:Mg, in that order, on a (0001)-oriented patterned sapphire substrate with a 30-nm-thick GaN buffer layer that had been grown by metal-organic chemical vapor deposition (MOCVD). LED chips were then fabricated. First, the wafer was etched using the inductively coupled plasma reactive ion etching (ICP-RIE) method until the sapphire substrate formed a mesa array of size 20 mil × 40 mil. Then, the surface of the p-type GaN layer was partially etched using the ICP-RIE method until the n-type GaN layer was exposed. Thereafter, a reflective conductive ITO/Ag (100 nm/500 nm) film layer was formed on the p-type GaN layer. Cr/Au (50 nm/2000 nm) electrodes were formed simultaneously on the ITO/Ag film and the exposed n-type GaN layer on the front of the wafer. The thickness of the wafer was lapped down to approximately 120 μm. GaN-based LEDs were mounted on a silicone plate (OE-6650) that was used as the temporary substrate, and this silicone substrate was heated to 120 °C for 60 min to cure LED chips. Subsequently, a KrF excimer laser with a wavelength of 248 nm and a pulse energy of 100~700 mJ was used to separate the sapphire substrate from the epitaxial LED structure. The laser beam was incident from the polished back surface of the sapphire substrate onto the sapphire/GaN epilayer interface. This process is the so-called laser lift-off (LLO) process. The sapphire/GaN epilayer interface absorbed the energy of the KrF laser beam, producing a high temperature such that the GaN buffer layer between the sapphire substrate and the GaN epilayer dissolved and the GaN epilayer was stripped from the sapphire substrate. The GaN-based thin-film LEDs (TF-LEDs) were thus formed. Finally, the GaN-based TF-LEDs were transferred onto a ceramic substrate, and then, the silicone temporary substrate was removed using acetone.

**Fig. 1:**
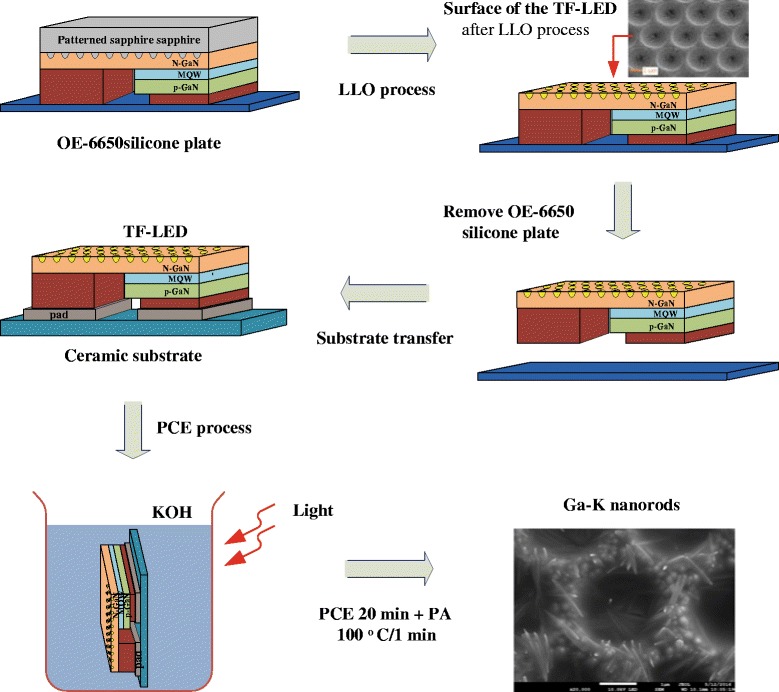
Preparation of GaN-based TF-LEDs on ceramic substrates

The GaN-based TF-LEDs were etched by dipping them in a KOH solution under illumination from a 300-W halogen lamp; this process is the so-called photo-assisted chemical etching (PCE) process. The GaN-based TF-LEDs were dipped in 3 M KOH solution for the 10, 20, or 30 min. The surface morphologies that were obtained following various periods of etching were observed. The PEC process was carried out using 3 M KOH and various etching times at 40 °C under illumination. Finally, photo-enhanced post-annealing (PA) was performed at 100 °C for 1 min to form Ga-K nanorods on the surface of the n-GaN layer. Figure 1 schematically depicts the procedure for preparing GaN-based TF-LEDs on ceramic substrates. The photograph on the bottom-right of Fig. *1* is a top-view SEM image of the surface of the GaN-based RF-LED following the PEC process and the PA process. The chip size was 20 mil × 40 mil. The current-voltage (*I*-*V*) and optical characteristics of the GaN TF-LED following the PCE process were measured.

## Results and discussion

**Fig. 2:**
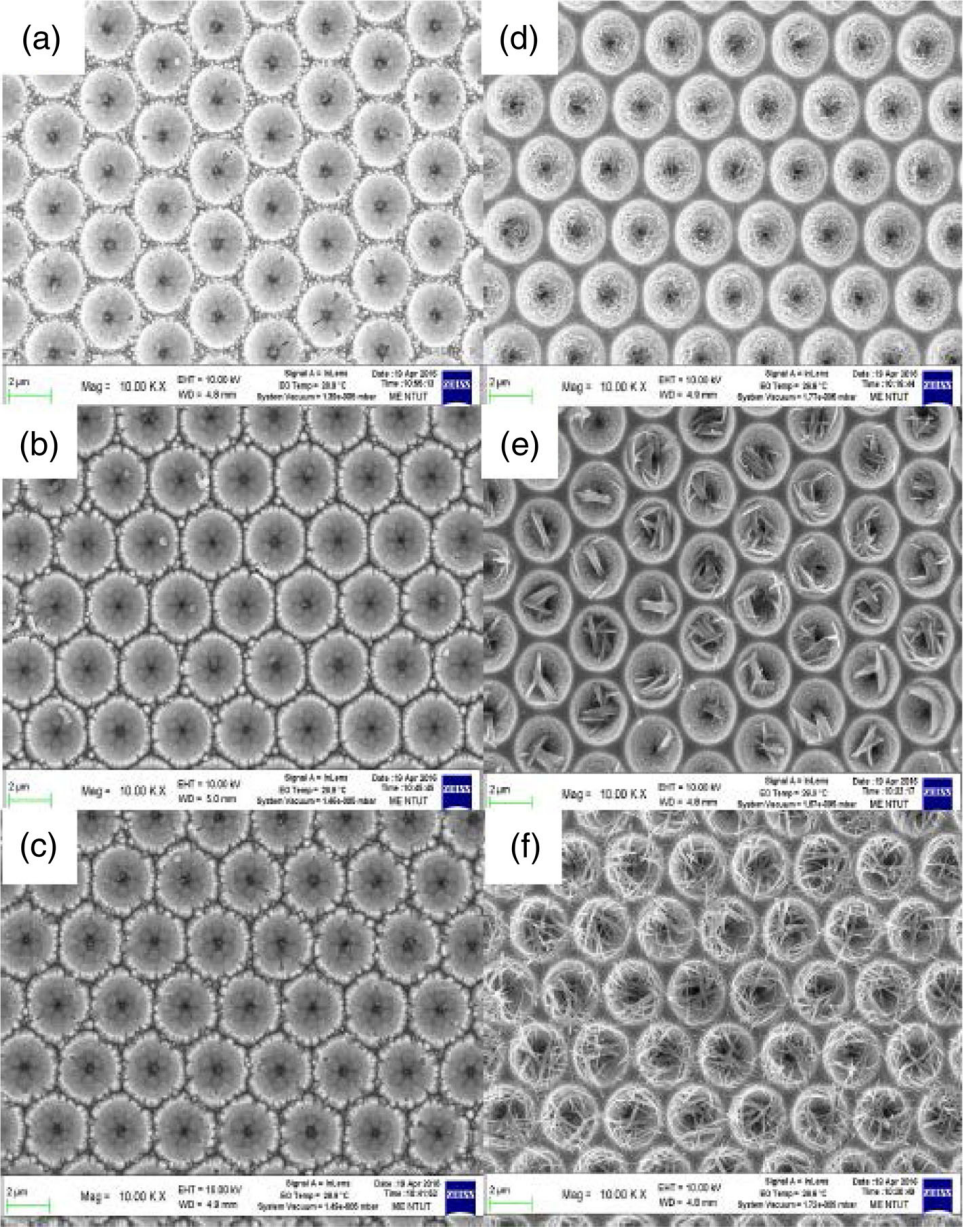
**a**–**c** Top-view SEM images of GaN-based TF-LEDs following PCE for 10, 20, and 30 min. **d**–**f** Top-view SEM images of GaN-based TF-LEDs following PCE for 10, 20, and 30 min and post-annealing at 100 °C for 1 min

The degradation in the light extraction efficiency of GaN-based LEDs following the LLO process is observed. A roughing process was performed after the LLO process to enhance the light extraction efficiency. Figure 2a, c presents top-view SEM images of the GaN-based TF-LEDs after the PCE process with various etching times without post-annealing. The hexagonal pyramid structure was not obvious after etching for 10 min, but was clearly present after etching for 20 or 30 min. Figure 2d–f presents top-view SEM images of Ga-K nanorods that formed on the surface of the n-GaN epilayer. They were formed by the PCE process for 10, 20, and 30 min, respectively, followed by post-annealing treatment at 100 °C for 1 min. The density of the nanorods increased with the etching time. The nanorods were formed by post-annealing (PA), which caused the Ga in the KOH solution to react with K, and re-crystallize on the surface of the n-GaN layer.

**Fig. 3:**
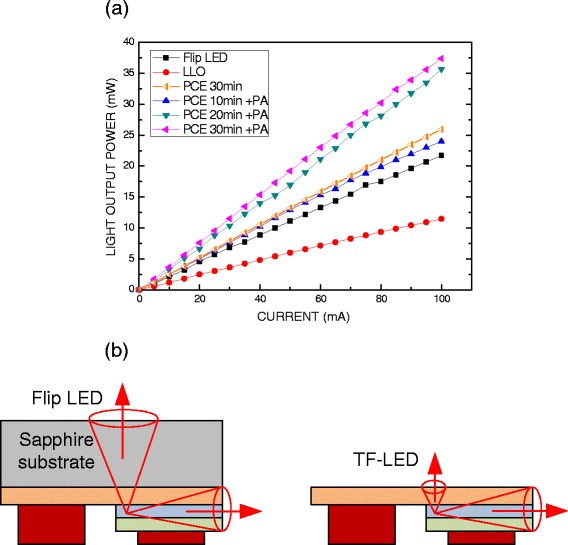
**a** Light output power of GaN-based LEDs with and without LLO, PCE, and post-annealing processes. **b** Schematic cross section, showing escape of light from top surface and sidewall of LEDs

Figure 3a plots the light output power as a function of the injection current for the GaN-based TF-LEDs with and without LLO, PCE, and post-annealing (PA). The light output power of all of the samples increased linearly with the injection current, suggesting the lack of a heat effect in all of the GaN-based LEDs in this work with injection currents in the range 0–100 mA. At an injection current of 100 mA, the light output power of the GaN-based flip LEDs that had not undergone the LLO process was around 21.7 mW. The LLO process reduced the light output power of the GaN-based TF-LED to 11.5 mW because it reduced the amount of light that escaped from the top surface of the GaN-based TF-LED, as shown in Fig. 3b.

Therefore, GaN-based TF-LEDs must undergo a surface roughing process to improve their light output power. The GaN-based TF-LEDs herein were placed in a beaker that contained KOH solution for various periods to produce a rough surface, as presented in Fig. 2a–c. The light output power of the GaN-based TF-LEDs following the PCE process for 30 min at an injection current of 100 mA was 25.9 mW, which was around 19.4% greater than that of flip LEDs.

Photo-enhanced PA at 100 °C was conducted for 1 min after the PCE process. Numerous nanorods are observed on the surface of the n-GaN epilayer, as presented in Fig. 2d–f. These nanorods were crystalline Ga-K, as revealed by EDX spectroscopy. The TF-LED that underwent the PCE process for 30 min and the PA process had the highest light output power, which was 37.4 mW at an injection current of 100 mA, which was around 72% more than that of the GaN-based TF-LEDs that had not undergone PCE and PA following the LLO process. In contrast, the light output power of the TF-LED that underwent the PCE process for 30 min and not underwent the PA process was 25.94 mW at an injection current of 100 mA. Therefore, nanorods on the surface of the LEDs are estimated to yield a light output power that is approximately 44% greater than that achieved with hexagonal pyramids, revealing better light scattering and light extraction efficiency.

**Fig. 4:**
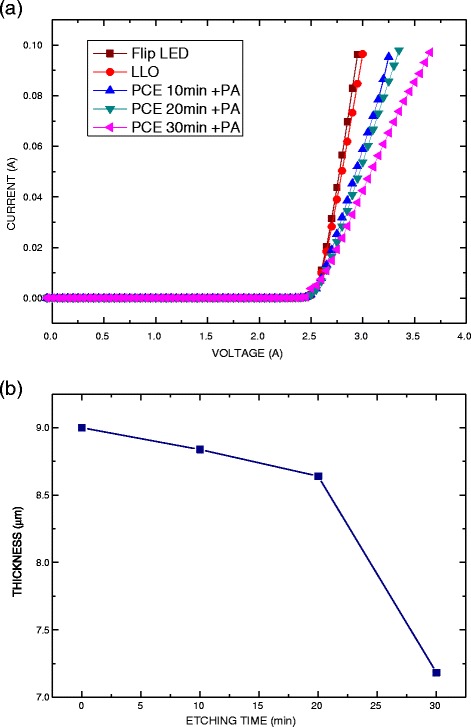
**a** *I*-*V* characteristics of GaN-based LEDs with and without LLO, PCE, and post-annealing processes. **b** Thickness of n-GaN epilayer after etching for various times

Figure 4a plots the *I*-*V* characteristics on a linear scale. The forward bias of the GaN-based flip LED that had not undergone the LLO process was approximately 2.95 V at an injection current of 100 mA. After the LLO process, the GaN-based TF-LED was formed. The forward bias increased slightly to 3.0 V because the contact resistance between the n-GaN epilayer and electrode was increased by residues of Ga_2_O_3_ on the surface of the n-GaN epilayer [21]. GaN was dissolved into Ga and N_2_ because a high temperature was generated by the KrF laser. Then, the metal Ga was oxidized and formed Ga_2_O_3_ on the surface of the n-GaN epilayer. The GaN-based TF-LEDs were underwent PCE as a surface roughing process to enhance their light output power. The forward bias of the GaN-based TF-LEDs following PCE and PA processed increased from 3.25 to 3.65 V as the etching time increased for 10 to 30 min because the thickness of the n-GaN epilayer was reduced, increasing the resistance effect of the GaN-based TF-LEDs, as shown in Fig. 4b.

## Conclusions

In summary, a process for the fabrication of a thin-film LED using laser lift-off and surface roughening processes was presented. GaN-based TF-LEDs were etched using a photo-assisted chemical (PEC) process to remove the residues from the surface. At an injection current of 100 mA, the light output power of the GaN-based flip LEDs that had not undergone the LLO process was approximately 21.7 mW. The LLO process reduced the light output power of the GaN-based TF-LED to 11.5 mW. The TF-LED that underwent the PCE process for 30 min and the PA process had the highest light output power of 37.4 mW at an injection current of 100 mA, which was approximately 72% greater than that of GaN-based TF-LEDs that had not undergone PCE or PA following the LLO process. The Ga-K nanorods on the surfaces of the GaN-based TF-LEDs that were prepared by the PCE process and PA process improved their light output power.

## Abbreviations

*I*-*V*: Current-voltage
LLO: Laser lift-off
PA: Post-annealing
PCE: photo-assisted chemical etching
SEM: scanning electron microscope
TF-LED: Thin-film light-emitting diode
